# Correction: Silver/silver halide supported on mesoporous ceria particles and photo-CWPO degradation under visible light for organic compounds in acrylonitrile wastewater

**DOI:** 10.1039/d4ra90005g

**Published:** 2024-01-24

**Authors:** Guozheng Zhao, Qingwei Tan, Changbo Li, Liyan Shang, Daihang Zhang, Xuanxuan Lu, Feng Qiu

**Affiliations:** a Liaoning Petrochemical University Fushun Liaoning 113001 China lnpulcb@126.com

## Abstract

Correction for ‘Silver/silver halide supported on mesoporous ceria particles and photo-CWPO degradation under visible light for organic compounds in acrylonitrile wastewater’ by Guozheng Zhao *et al.*, *RSC Adv.*, 2021, **11**, 26791–26799, https://doi.org/10.1039/D1RA04465F.

The authors regret the omission of a funding acknowledgement in the original article. This acknowledgement is given below.

The project was funded by National Water Pollution Control and Management Technology Major Projects (2012ZX07202-002); Science and Technology Research Project of Liaoning Provincial Department of Education (L2020020, L2020003); and the Talent Scientific Research Fund of LNPU (no. 2020XJJL-015).

The Royal Society of Chemistry apologises for these errors and any consequent inconvenience to authors and readers.

## Supplementary Material

